# Effects of acupuncture for Bell’s palsy patients in the acute phase and its impact on facial nerve edema: a study protocol for a randomized, controlled trial

**DOI:** 10.3389/fneur.2024.1327206

**Published:** 2024-04-16

**Authors:** Zhidan Wang, Jie Zhang, Zhen Zhang, Yue Liu, Shuang Ren, Hao Sun, Di Meng, Ruoshi Liu, Yang Zhang

**Affiliations:** ^1^Department of Traditional Chinese Medicine, The First Hospital of China Medical University, Shenyang, China; ^2^Department of Ultrasound, The First Hospital of China Medical University, Shenyang, China; ^3^Department of Clinical Epidemiology, The First Hospital of China Medical University, Shenyang, China; ^4^Department of Geratology, The First Hospital of China Medical University, Shenyang, China

**Keywords:** Bell’s palsy in the acute phase, acupuncture, electroacupuncture, edema of the facial nerve, randomized clinical trial

## Abstract

**Background:**

Bell’s palsy is an acute peripheral facial neuropathy, which is one of the most common causes of facial palsy of lower motor neurons. Facial nerve swelling is commonly observed in Bell’s palsy. Acupuncture therapy has been widely used in the treatment of Bell’s palsy. However, whether acupuncture can be effectively used in the acute stage is still controversial. There are no clinical trials conducted previously to evaluate the effect of acupuncture on facial nerve edema in Bell’s palsy patients. The study aims to evaluate the potential efficacy of different acupuncture modalities on Bell’s palsy patients in the acute phase, its effect on facial nerve edema, and to preliminarily explore its possible mechanism.

**Methods and analysis:**

In this randomized, controlled trial, 165 Bell’s palsy patients with unilateral onset within 3 days will be recruited and randomly assigned to either the electroacupuncture group (*n* = 33), the acupuncture group (*n* = 33), the sham acupuncture group (*n* = 33), the blank control group (*n* = 33), or the acupuncture control group (*n* = 33) in a 1:1:1:1:1 ratio. The participants will receive 4 weeks of treatment and 8 weeks of follow-up. The five groups of participants will receive the following treatments: A: Electroacupuncture + Medication (prednisone acetate tablets, mecobalamin tablets, and vitamin B1 tablets); B: Acupuncture + Medication; C: Sham Acupuncture + Medication; D: Medication only; and E: Acupuncture only. The primary outcome will be the effectiveness rate of different acupuncture modalities in improving facial nerve function after the intervention period. The secondary outcomes will be the recovery speed, the diameter of the facial nerve, the echo intensity and thickness of facial muscles, blood flow parameters of the facial artery, the serum inflammatory level, safety evaluation, and adverse events. Preliminary exploration of its mechanism of action occurs through inflammation and immune response. The difference between groups will be assessed using repeated measure analysis of covariance (ANCOVA) and trend chi-square.

**Discussion:**

The trial will evaluate the efficacy and facial nerve edema of acupuncture for Bell’s palsy patients in the acute phase and preliminarily explore its possible mechanism. The results thus may provide evidence for clinical application.

**Clinical trial registration:**

https://www.chictr.org.cn/bin/project/edit?pid=133211, identifier ChiCTR2100050815.

## Introduction

Bell’s palsy is an acute peripheral facial neuropathy, which is one of the most common causes of facial palsy of lower motor neurons (1). Bell’s palsy is defined as an acute unilateral facial nerve palsy or paralysis, with an onset time of less than 72 h, and has unknown etiology. It accounts for 50% of all facial nerve paralysis cases (2), and the incidence rate has been reported between 11.5 and 53.3 per 100 thousand persons (3). The facial nerve is the longest nerve in the human body that traverses through the bone canal, and thus, its anatomical characteristic makes it easy to cause local blood circulation obstacles after an injury and cause nerve edema. The etiology of Bell’s facial palsy remains unclear. The only confirmed finding is that the inflammation and edema of the facial nerve in the narrow styloid foramen can lead to the compression of the facial nerve canal. Facial nerve swelling is commonly observed in Bell’s palsy and has been reported during decompression surgery (4, 5). The different stages of facial palsy are defined as follows: acute phase: 1–7 days; resting phase: 8–20 days; and recovery period: 21–70 days (6). Acupuncture has been widely used in the treatment of Bell’s palsy and has achieved satisfactory results to date (7). Several clinical and laboratory studies have found that acupuncture can effectively promote the recovery of facial nerve injury, effectively shorten the course of the disease, and reduce various complications (8, 9). Especially in Asian countries, such as Korea, Japan, and China, there are a large number of patients who have been managed by acupuncture therapy as the initial treatment. The findings of clinical practice in our department and some prior studies have proved that early use of acupuncture in acute Bell’s palsy can significantly slow down the progress of facial nerve injury, improve the curative effect, shorten the clinical recovery time, and thus reduce the sequelae (10, 11). “Clinical Guidelines for diagnosis and treatment of Traditional Chinese Internal Medicine” and other guidelines made a Grade A recommendation that acupuncture should be involved in the treatment of facial palsy as soon as possible, and the patients with mild or severe facial palsy may be treated with any one of acupuncture, western drugs, or acupuncture combined with drugs (12). However, some experts usually do not accept acupuncture as the recommended strategy for the management of the acute stage of facial nerve injury, possibly because they are afraid that the potential application of acupuncture in the acute stage can aggravate nerve edema (2). However, Fang et al. have used thick needle therapy to treat acute ischemic facial nerve injury in rats. It was found that the swelling degree of the facial nerve in the treatment group was markedly lower than the control group on days 1, 3, 5, and 7, which promoted the regression of the facial nerve edema in the bone canal (13). However, there is no prior report on the potential effects of acupuncture for facial edema in patients with acute Bell’s palsy.

For the objective quantification of the facial nerve edema, enhanced magnetic resonance imaging (MRI) can be effectively used to observe facial nerve ischemia and edema, but it is actually contrast-enhanced rather than physical nerve swelling, which is costly, time-consuming, and associated with many limitations (14, 15). Neuroultrasound is a non-invasive examination method to objectively describe facial nerve edema. It can facilitate real-time image acquisition to describe the structural changes of the nerve and has the advantages of cheap price, easy access, and bedside use (16). In addition, pathological studies have confirmed that local ischemia of the affected lateral nerve in patients with Bell’s palsy could be often observed. Because the direction of blood flow of the facial nerve is mainly from proximal to distal, the microcirculation of facial skin might also get adversely affected after Bell’s palsy attack (17). For example, Yin et al. (18) observed the potential changes in the blood flow at the facial acupoints of patients with Bell’s palsy by Doppler ultrasound and found that there were significant differences in the average velocity, final diastolic velocity along the maximum velocity curve, peripheral resistance index at the four facial acupoints between the affected side and the healthy side, and between patients with different facial palsy degrees. Therefore, color Doppler ultrasound can dynamically and quantitatively monitor the changes in the facial artery blood flow parameters in patients with Bell’s facial palsy during acupuncture treatment, which can be used as an important basis for the analysis of observed curative effects.

At the same time, ultrasound can also evaluate the muscle condition of patients with facial palsy by observing the area, depth, and echo intensity of facial muscles. Volk et al. used quantitative muscle ultrasound to assess the muscle area, thickness, and echo intensity of 2 masticatory muscles and 6 facial muscles in 20 patients with chronic facial palsy. The results showed that the lateral muscles of the paralyzed side decreased significantly, and the echo intensity of other facial muscles increased markedly except that of the frontalis and orbicularis oculi muscles (19).

Therefore, we have designed a randomized controlled trial (RCT) to examine the possible efficacy of different acupuncture modalities on patients with Bell’s palsy in the acute phase and the improvement of facial nerve edema using appropriate randomization and rigorous blinding conditions. The objectives of this trial are as follows:

1. To evaluate the efficacy of different acupuncture modalities on patients with Bell’s palsy in the acute stage;
2. To assess whether different acupuncture modalities can effectively relieve facial nerve edema in patients with Bell’s palsy in the acute stage;
3. To analyze whether different acupuncture modalities can significantly improve facial blood circulation and muscle activity in patients with Bell’s palsy in the acute stage;
4. To provide reference for the timing of different acupuncture modalities intervention;
5. To explore the mechanisms that can contribute to both inflammatory and immune responses.

## Methods and analysis

### Study design

**Figure 1:**
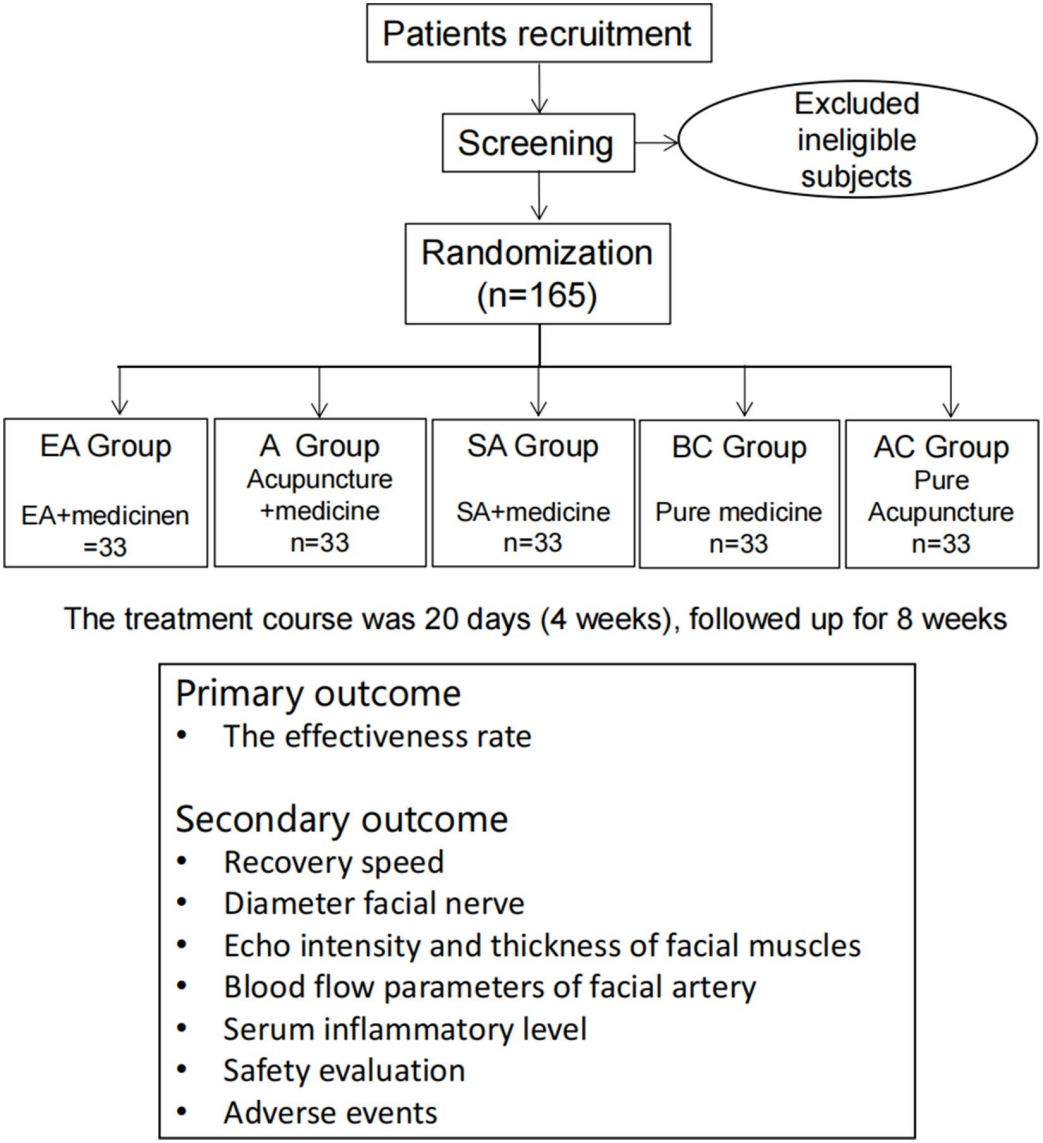
Flow chart of the planned study. EA: electroacupuncture; A: acupuncture; SA: sham acupuncture; BC: blank control; and AC: acupuncture control.

This will be a single-center, randomized, controlled clinical trial. The study has been approved by the ethics committee of the First Affiliated Hospital of China Medical University (CMU1H). It will be conducted from October 2021 to December 2025 in the Traditional Chinese Medicine (TCM) Department, Emergency Department, Ultrasound Department, and Neurology Department of CMU1H, which is the largest comprehensive hospital in northeast China. All the participants will be informed about the study and sign written informed consent before participating in the trial. Eligible patients will then be randomly divided into the electroacupuncture group (EA + medicine), the acupuncture group (A + medicine), the sham acupuncture group (SA + medicine), the blank control group (pure medicine), and the acupuncture control group (pure acupuncture) at a ratio of 1:1:1:1:1. The first three groups will be treated with electroacupuncture, acupuncture, or sham acupuncture five times a week based on medicine therapy. The blank control group will be treated with medicine only, and the acupuncture control group will be treated with acupuncture five times a week without medicine-based treatment. The treatment course will last for 4 weeks and thereafter follow up for 8 weeks. This trial is designed in strict accordance with the Consolidated Standards of Reporting Trials (CONSORT) statement and the recommendations of Standards for Reporting Intervention in Controlled Trials of Acupuncture (STRICTA) (20) and will be reported based on the Standard Protocol Items: Recommendations for Interventional Trials (SPIRIT) 2013 Checklist (21). The trial flow chart is shown in Figure 1, and the study design schedule is shown in Table 1. Supplementary material S1 contains the complete spirit list.

**Table 1:** The designed schedule for the data collection.

Time point	Screening period	Intervention period					Follow-up period	
	Day 0	Day1	Day 5 (Week1)	Day 10 (Week2)	Day 20 (Week4)	Once daily until discharge	Week 8 ± 3 days	Week 12 ± 3 days
Enrolment								
Informed consent	×							
Eligibility screening	×							
Randomization	×							
Allocation	×							
Interventions								
The electroacupuncture group	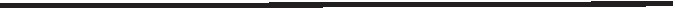							
The acupuncture group	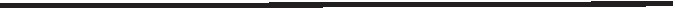							
The sham acupuncture group	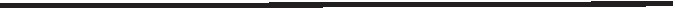							
The blank control group	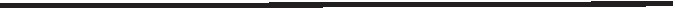							
The acupuncture control group	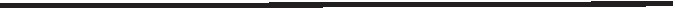							
Assessments								
SFGS	×		×	×	×	×	×	×
HBGS	×		×	×	×	×	×	×
The diameter of facial nerve	×		×	×	×		×	×
Echo intensity and thickness of facial muscles	×		×	×	×		×	×
Blood flow parameters of the facial artery	×		×	×	×		×	×
Therapeutic mechanism index	×		×	×	×		×	×
Safety index	×		×	×	×			
Adverse events			×	×	×	×		
Success of blinding					×			

The treatment course will last for 20 days (4 weeks) and thereafter follow up for 8 weeks. First, the subjects will be informed of informed consent and screening. Then, they will be randomized into five different groups. The four groups related to acupuncture will be treated with acupuncture 5 times a week for a total of 4 weeks (20 days); the blank control group will receive the medicine therapy (see “Intervention” for details). The clinical data will be evaluated at baseline, days 5, 10, and 20 (weeks 1, 2, and 4), and weeks 8 and 12 of treatment. The Sunnybrook facial grading system (SFGS), the House-Brackmann facial nerve grading system (HBGS), and adverse events will be evaluated daily during treatment. During treatment, adverse events will be recorded in the Case Report Form (CRF) at any time.

### Participants

#### Recruitment strategy

The participants of the study will be recruited from the outpatient, emergency, and ward departments of the CMU1H using WeChat advertising and posters. Interested patients can enquire from the clinician about the various details of the project through telephone or direct meetings. The investigator will also describe the purpose, content, research process, benefits, and risks of the study to the participants in detail. The researchers will screen the patients according to the study protocol, and eligible patients will then be recruited for the clinical trials.

#### Inclusion criteria

The study includes the following inclusion criteria:

1. Participants who aged between 18 and 70 years;
2. Participants who meet the diagnostic criteria for Bell’s palsy, HBGS should be graded from II to VI, and the total score of SFGS should be ≤89;
3. The onset is less than or equal to 3 days;
4. The onset is unilateral; and
5. Participants who agreed to participate in the investigation and sign the written informed consent.

#### Exclusion criteria

Participants who will meet any of the following conditions will be excluded:

1. Participants who have otitis media, mastoiditis, labyrinthitis, mumps, and other complications of peripheral facial palsy;
2. Participants who have a prior diagnosis of peripheral facial palsy caused by posterior fossa lesions such as acoustic neuroma, skull base meningitis, intracranial metastasis of cancer, and multiple sclerosis;
3. Participants diagnosed with the central facial palsy;
4. Participants diagnosed with Hunt’s syndrome;
5. Participants with serious cardiovascular and cerebrovascular diseases, diabetes, hypertension, serious primary diseases of the liver, kidney, lung, and blood system, malignant tumors, ulcers of the digestive system, and bleeding tendencies are expected to fail to complete the test;
6. Participants who might be enrolled in other clinical trials within 1 month; glaucoma patients; and pregnant or lactation women or patients with severe allergic conditions. Those who have neurological, mental illness, illiteracy, or poor compliance in the screening process will not be eligible to fill in the questionnaire.

### Sample size

The calculation of the sample size is based on the effective rate of acupuncture in the treatment of patients with Bell’s palsy in the acute stage. According to the previous studies (22, 23) and pre-experimental analysis of the research group, it is estimated that the effective rate of the EA group is 98%, the A group is 95%, the AC group is 85%, the SA group is 74%, and the BC group is 70%. We used PASS 15 software to calculate and set α = 0.05 and β = 0.1, and the results showed that the total sample size was at least 147 cases. It was observed that based on the ratio of the sample size of 1:1:1:1:1 in each group and considering the loss of follow-up rate of 10%, an optimal sample size of 33 participants should be recruited in each group. Therefore, a total of 165 participants should be recruited for this RCT.

### Randomization

A random sequence will be generated based on block randomization by an independent research assistant using the software SPSS 26.0. The various eligible participants will be randomly divided into five groups with a 1:1:1:1:1 ratio. The treatment allocation codes will be enclosed by an independent researcher in sequentially numbered opaque envelopes until the first treatment schedule. To minimize coding interruptions, the chief investigator and result evaluators will also turn a blind eye to the treatment allocation.

### Blinding

The blinding method will not be fully suitable for both patients due to the obvious difference in types of intervention between the BC group and the other groups. However, laboratory technicians, ultrasound doctors, and statisticians will be completely blinded. At the same time, this trial will be blinded to recruited patients, the acupuncturists, data collectors, assessors, and statisticians between the other four groups. Patients will be treated in separate rooms or a curtain will be used to cover the bed, while patients will be wearing an eye mask. In the sham acupuncture group, the acupoints will be on the face and the back of limbs, and the operation experience will be consistent with that of acupuncture.

### Interventions

The interventions will be combined with the guidelines (3) published by the American Academy of Neurology (AAN) and the American Academy of Otolaryngology-Head and Neck Surgery Foundation (AAO-HNSF) in 2013 and the preliminary experiments of this study (2). Acupuncture will be performed by three different experienced acupuncturists in accordance with STRICTA (19). The subjects in each group, except the blank control group, will receive either acupuncture, electroacupuncture, or sham acupuncture treatment 5 times per week for 4 weeks. Except for the acupuncture control group, patients in each group will receive the following medications. Prednisone acetate tablets will be administered orally at 30 mg per day for 5 days, then it will be reduced by 5 mg per day until discontinued for 10 days (Sinopharm, Rongsheng Pharmaceutical Co. LTD., Jiaozuo, China); Mecobalamin tablets of 0.5 mg will be orally administered, 3 times a day (Eisai (China) Pharmaceutical Co., LTD., Jiaozuo, China); and Vitamin B1 tablets of 10 mg will be administered orally 3 times a day (Fuzhou Haiwang Fuyao Pharmaceutical Co., LTD., Fuzhou, China). During the study, subjects will not be allowed to use traditional Chinese medicine treatment methods other than those prescribed medications to treat the disease (e.g., traditional Chinese medicine preparations, tuina therapy, etc.). The participants can withdraw from the trial for any reason and at any point in time. Researchers can remove participants from the trial with any of the following conditions: (1) the legal representative of the patient requested to withdraw from the study, (2) serious adverse events, and (3) death.

#### The acupuncture group and the acupuncture control group

The disposable sterile acupuncture needles will be used (0.25 × 40 mm, Suzhou Acupuncture Supplies Co., LTD., China). In all sessions, patients will be treated with eight core acupuncture points and four additional acupoints. The core acupoints will be BL02 (Cuanzhu), GB14 (Yangbai), ST2 (Sibai), SI18 (Quanliao), ST4 (Dicang), ST6 (Jiache), LI4 (Hegu), and LR3 (Taichong), and the additional acupoints can be chosen from the following points: middle ditch askew plus DU26 (Shuigou); nasolabial fold becomes shallow add LI20 (Yingxiang); eyelid closure difficulty plus EX-HN5 (Taiyang); and pain behind the ear mastoid process Ex-HN17 (Yifeng). All acupoints will be carefully selected according to the clinical experience and the literature. After the acupuncture operation, the needle will be retained for 30 min before removal. The treatment will be performed once a day, 5 times as a course of treatment, with 2 days rest among the courses, for a total of 4 courses (weeks). The acupuncture group will be combined with the medicine therapy based on acupuncture treatment. The acupuncture control group will be treated with acupuncture only. The acupuncture points and methods are summarized in Table 2.

**Table 2:** Acupuncture points and methods.

Acupoint types	Acupuncture point	Direction	Depth (mm)	Electroacupuncture point
Core acupoints	BL02 (Cuazhu, affected side)	Oblique stab in the direction of the eyebrow arch	3–10	No
Core acupoints	GB14 (Yangbai, affected side)	Downward inclined stab	3–10	Yes
Core acupoints	ST2 (Sibai, affected side)	Perpendicular to the skin	3–10	No
Core acupoints	SI18 (Quanliao, affected side)	Perpendicular to the skin	3–10	Yes
Core acupoints	ST4 (Dicang, affected side)	Transversely toward ST6	3–10	Yes
Core acupoints	ST6 (Jiache, affected side)	Transversely toward ST4	3–10	Yes
Core acupoints	LI4 (Hegu, healthy side)	Perpendicular to the skin	3–10	No
Core acupoints	LR3 (Taichong, healthy side)	Perpendicular to the skin	3–10	No
Additional acupoints	DU26 (Shuigou)	Perpendicular to the skin	3–10	No
Additional acupoints	LI20 (Yingxiang, affected side)	Oblique to the nasal root along the nasolabial groove	3–10	No
Additional acupoints	EX-HN5 (Taiyang, affected side)	Lateral epicanthic spur	3–10	No
Additional acupoints	Ex-HN17 (Yifeng, affected side)	Perpendicular to the skin	3–10	No

#### The electroacupuncture group

The acupuncture points and the treatment course of this group will be the same as those of groups A and AC, and four acupoints will be connected to an electroacupuncture device and treated with electroacupuncture. The needles on GB14 (Yangbai), SI18(Quanliao), ST4 (Dicang), and ST6 (Jiache) will be connected to the SDZ-II Acupuncture Stimulating Instruments (Suzhou Medical Supplies Factory Co., LTD., China) with 2-Hz frequency and the varying amplitude according to the comfort of the participants, ranging from 2 to 5 mA to enhance the sensation of acupuncture. The needles will be kept for 30 min and then removed.

#### The sham acupuncture group

Patients in this group will be treated with sham acupuncture at the following five non-meridian and non-acupoint stimulation points, and the specific location is shown in Table 3. The location of acupoints can be avoided by referring to the 2006 National Standard of the People’s Republic of China (GB/T 12346-2006) *name and location of acupoints*. Patients will be placed in the supine position, their skin will be disinfected, and the Streitberger placebo needle (0.30 × 30 mm) will be used (24). When the needle is attached to the skin through a plastic ring, the patient might feel a tingling sensation, mimicking a puncture to the skin. However, when the needle is pressed against the skin, it does not penetrate the skin but instead retracts into the handle. The frequency, course, and time of acupuncture will be the same as those of the acupuncture group.

**Table 3:** The location of stimulation points in the sham acupuncture group.

Stimulation point	Direction	Affected/Healthy side
Stimulation point 1	The midpoint of the line between GB14 (Yangbai) and ST8 (Touwei)	Affected side
Stimulation point 2	The midpoint of the line between ST05 (Daying) and ST4 (Dicang)	Affected side
Stimulation point 3	The midpoint of the line between ST07 (Xiaguan) and SI18 (Quanliao)	Affected side
Stimulation point 4	The midpoint of the connection between the styloid process of the ulna and the olecranon	Healthy side
Stimulation point 5	The midpoint of the line between EX-HN5 (Taiyang) and SP5 (Shangqiu)	Healthy side

#### The blank control group

Except for the medicine therapy, no electropuncture, acupuncture, or sham acupuncture will be carried out during the whole experiment in this group.

### Outcome measures

The outcome evaluation will be conducted at 6 time points, including the baseline, days 5, 10, and 20, and weeks 8 and 12 of treatment. In addition, SFGS and HBGS will be evaluated before each treatment to evaluate the recovery rate. Safety measures will be assessed at the baseline and day 20 of the treatment. The evaluation schedule is shown in Table 1.

#### Primary outcome measures

The primary outcome is the effectiveness rate after the intervention period, which will be evaluated immediately at the end of treatment on the 20th day. Combined with the results of SFGS and HBGS, the efficacy evaluation criteria have been formulated according to the *Evaluation and efficacy standard of Integrated Chinese and Western Medicine for peripheral facial nerve palsy (draft)* by Yang et al. (25) as shown in Table 4. The effectiveness rate is calculated by dividing the sum of the number of cured, efficacious, and effective patients in each group by the number of patients in each group. The Sunnybrook facial grading system (SFGS) is composed of three distinct areas that generate a comprehensive score describing the overall static and dynamic state of the face. Final score = free movement points – static points – linkage points. SFGS score can range between 0 and 100, and the higher the score is, the better facial nerve function (26). The House-Brackmann facial nerve grading system (HBGS) is a scale for assessing the severity of Bell’s palsy, which can potentially classify facial nerve injury into six grades (27). Grade I indicates normal function, grade II mild dysfunction, grade ≤ IV moderate palsy, and grade ≥ V severe palsy (27). SFGS and HBGS scores will be evaluated prior to each treatment until the end of the study and during the follow-up periods of 8 and 12 weeks of treatment.

- Recovery speed: the patient’s recovery will be evaluated daily through SFGS and HBGS until the patient is assessed as “cure” according to the evaluation criteria for curative effect (Table 4). The period from baseline to this time is considered the recovery time. If the patient’s recovery time is shorter, it will be considered that the recovery speed is faster.
- The diameter of the facial nerve: bilateral nerve color Doppler ultrasonography will be conducted by a board-certified neurosonologist in the Department of Ultrasound of CMU1H. The diameter of the main trunk and five branches of the facial nerve on both sides of the face will be measured three times, and the average value will be taken.
- Echo intensity and thickness of facial muscles: the thickness and recovery strength of the frontalis muscle, the orbicularis oculi muscle, the orbicularis oris muscle, the depressor anguli oris muscle, the depressor labii inferioris, and the mentalis muscle will be measured using a diagnostic ultrasound system. The determination of maximum muscle thickness has been reported to be orthogonal to the direction of muscle fibers (19). Thus, gray analysis will be used to quantify the echo intensity of each muscle (19). The results of three independent measurements for each muscle will be averaged to minimize the differences.
- Blood flow parameters of facial artery: color Doppler ultrasound will be used to measure and record the systolic peak velocity (Vs), end-diastolic velocity (Vd), and resistance index (RI) of bilateral facial artery, inferior labial artery, and superior labial artery. All data will be measured three times and thereafter averaged.
- The serum inflammatory level: at the baseline; 5, 10, and 20 days of treatment; 8 and 12 weeks of treatment; the count of neutrophils, lymphocytes, platelets; and the levels of interleukin-6 (IL-6), interleukin-8 (IL-8), interleukin-10 (IL-10), and tumor necrosis factor-α (TNF-α) will be measured to assess the levels of immune inflammation.
- Safety evaluation: the various safety indicators will be measured at the baseline and the end of treatment, including blood routine (the count of neutrophils, lymphocytes, platelets, monocytes, and hemoglobin content), the liver function (alanine aminotransferase and aspartate aminotransferase), and the kidney function (blood urea nitrogen and serum creatinine).
- Adverse events: acupuncture can exhibit potential adverse events, such as pain, hematoma, and infection, and the participants will be clearly informed about all these adverse events before signing informed consent. We will also provide appropriate medical care for the commonly observed adverse reactions. Any adverse events will be recorded by CRF. In case of serious adverse events, treatment will be terminated, and a detailed report will be made to researchers and the ethics committee within 24 h of the occurrence. The ethics committee will make recommendations and decide whether the patient can continue the treatment. We will give them proper compensation for their medical expenses.

**Table 4:** Evaluation criteria for curative effects.

Curative effect	HBGS	SFGS
Cure	Grade I	≥ 90
Efficacious	Grade II	70–89
Effective	Grade III	50–69
Ineffective	Grade IV and above	< 50

### Data collection

The CRF will be completed by the clinical investigator. Clinical investigators will have to ensure accuracy, completion, and timely loading of data into CRF while preserving the original records. The completed CRF and other scales will be reviewed by the clinical supervisor and transferred to the data manager. To reduce shedding and increase study compliance, the investigator and data collector will remind the subjects about this procedure 1 day before the scheduled visit date.

### Data management

There will be two data administrators entering and proofreading the data to ensure accuracy. If any problem is detected with the data, the data supervisor will ask the researcher for clarification. After the study, the clinical researchers, data managers, and statistical analysts will carefully review the established database. After a blind audit and confirmation of the correctness of the established database, principal researchers and statistical analysts will lock the data. The locked data files will remain unchanged. The data will only be used for this specific research project.

### Confidentiality

All information collected during the study will be kept confidential and held by the investigator. The various researchers, members of the ethics committee, and relevant management departments have the right to review the information records of the participants to the extent permitted by the law. The personal information of the participants will not be independently disclosed in any research report or publications related to the project.

### Patients and public involvement

Patients and the public will not participate in the analysis and evaluation of the results of this study. The results will be disseminated through publication in peer-reviewed journals.

### Statistical analysis

The statistical analysis of this trial will use SPSS 26.0 software. To include the data of the participants who might withdraw later from the trial, all statistical analyses were based on the intention-to-treat population of all randomly assigned patients. Missing data will be analyzed using multiple imputations using the Markov chain Monte Carlo. The continuity data will be described as mean ± standard deviation (SD) or median and interquartile spacing. The classified data will be described with the frequency and percentage (*N*, %). The assessment of the difference between groups will use repeated measure analysis of covariance (ANCOVA) and trend chi-square, and the mixed effect model will also be used to evaluate the efficacy based on adjusting possible covariates. For results that require a comparison between multiple groups, Bonferroni will be used for multiple corrections. An interim analysis will be performed when half of the participants have completed the main outcome measurement.

### Quality control

The participants involved in the implementation will be trained in a unified manner, using unified recording methods and judgment standards. We will formulate and implement rigorous, detailed, and feasible relevant standard operating procedures (SOP), and clinical supervisors and the data supervisors will carefully supervise the whole process of investigation according to the SOP. The investigator shall accurately and carefully record all the contents in CRF according to the filing requirements of the case report form so as to ensure the authenticity and reliability of the case report form. All observations and findings in the trial will be verified to ensure the reliability of the data and to ensure that all conclusions in the clinical trial have been derived from the original data.

### Data monitoring and trial steering committee

The data and safety monitoring of this trial will be entrusted to the data monitoring committee (DMC) and the Data and Safety Monitoring Committee (DSMB) of CMU1H, which are independent of the sponsors and the research group and have no competitive interests. The main function of DMC will be to monitor the treatment and integrity of the trial data and conduct interim analysis to confirm whether the trial confirms the principles of this protocol. DSMB consists of five renowned experts in different fields and monitors the performance and safety of the trial every 6 months. It will have the right to obtain the various interim results of the trial, reveal a participant’s allocated intervention, and make the final decision on whether to terminate the trial.

### The modification of the protocol

Any modifications to the protocol will require the formal approval of the ethics committee of CMUIH and the Chinese clinical trial registry.

### Post-trial care

This study does not plan to provide any post-trial care.

#### Trial status

The version number of this protocol is 2.0, dated 1 June 2021. The clinical trial is currently underway. The recruitment started on 1 October 2021 and is scheduled to be completed by the end of 2025.

## Discussion

A number of studies have shown the effectiveness and safety of acupuncture in the treatment of Bell’s palsy, and acupuncture has been widely used in the non-drug alternative treatment of Bell’s palsy. However, it is still not clear whether acupuncture can be applied to patients in the acute phase, especially electroacupuncture.

The main controversy is whether acupuncture is beneficial to facial edema in the acute stage of Bell’s palsy (28, 29). To the best of our knowledge, this clinical trial is the first study planned to analyze the effect of facial edema in patients with Bell’s palsy in the acute phase by using facial nerve color Doppler ultrasound. The main purpose of this trial is to present a well-designed parallel randomized sham-control trial to evaluate the potential efficacy of different acupuncture modalities on acute Bell’s palsy patients and its effect on facial nerve edema. It also aims to preliminarily explore its mechanism through inflammatory and immune indicators to provide a reference for the selection of clinical acupuncture treatment opportunities.

First, we have designed two observation groups and three control groups. These groups have been allocated adequately to completely compare the effects of acupuncture, electroacupuncture, and medicine treatment on patients with Bell’s palsy in the acute phase. To determine whether real acupuncture or placebo effect might be responsible for the treatment, non-point and non-transdermal operation of overlapping blunt needles will be performed as sham acupuncture control.

Second, to achieve better therapeutic effects, we have chosen a semi-standardized acupoint selection scheme of core acupoints and additional acupoints according to the guidelines (12) and the clinical experience of acupuncturists in the department, and light stimulation will be given according to the principle of “shallow needling and less needling in acute stage.” For the selection of acupoints in the SA group, non-meridian and non-acupoint stimulation will also be selected to eliminate the interference of meridians and acupoints.

Third, the combination of HBGS and SFGS will be used as the evaluation standard of curative effect. HBGS is a scale for assessing the severity of Bell’s palsy, recommended by AAN and AAO-HNSF (2). HBGS has emerged as the most commonly used and reliable scale in clinics and has been widely used. However, its practicality is significantly limited due to observations of only large-scale effects. SFGS has gradually attracted the attention of researchers at home and abroad in recent years. A number of researchers have indicated that SFGS is the best scoring system for facial palsy and have recommended it as a supplement to the longitudinal grading score of HBGS (30, 31). The system is sensitive to the facial improvement evaluation and can display a higher value for follow-up (32). Therefore, the combination of HBGS and SFGS can be used to comprehensively evaluate the degree of facial paralysis and facial nerve functions.

Fourth, in addition to the diameter of the trunk, the acquisition of color Doppler ultrasound of the facial nerve also includes the diameter and echo intensity of the five branches to completely determine the situation of facial nerve edema. For the facial blood flow information, Vs, Vd, and RI of the facial artery will be collected by ultrasonic Doppler to observe the potential effect of acupuncture on facial blood flow in patients with Bell’s palsy in the acute phase. The thickness and echo intensity of five different facial muscles, such as the frontal muscles, will be measured to observe the effect of acupuncture on the facial muscles in patients. This will aid us to observe the possible improvement of facial blood circulation and muscle activity in patients with acute Bell’s palsy by acupuncture.

Fifth, although the pathogenesis of Bell’s palsy is not completely clear, inflammation plays an important role in this condition. Most researchers agree that an abnormal immune response caused by viral infection is closely related to the occurrence and development of facial nerve palsy. Thus, strategies to control the degree of neuroinflammatory response can prove to be beneficial to the outcome of the disease by functionally regulating the immune function of the body (33). The neutrophil-to-lymphocyte (NLR) ratio has been recognized as a new potential marker to determine inflammation, which is routinely measured in peripheral blood and plays a vital role in evaluating Bell’s facial palsy (34, 35). The guidelines for the management of acute Bell’s palsy, published by the French Society of ENT and Head and Neck Surgery (SFORL) in 2020, also recommended that a complete blood count screen for neutrophil/lymphocyte ratios should be conducted, as elevated levels might suggest a poor prognosis (8). The measurements of the serum samples from patients with Bell’s palsy showed significantly higher levels of IL-6, IL-8, and TNF-α in patients with Bell’s palsy compared to controls (36). In addition, a previous study aimed at understanding the mechanism of EA in the treatment of patients with lumbar disc herniation, which is also a neuro edema disease, and found that EA can effectively reduce the levels of IL-2, TNF-α, and IL-6 contents (37).

Meanwhile, this trial might face some potential challenges that must be solved. First of all, since patients with Bell’s palsy will be recruited within 3 days of onset, the number of patients may be small. Thus, we have made widespread use of the internet and community publicity to increase awareness, and at the same time, we will provide free examination and treatment for patients to draw attention from patients. Second, to completely ensure blindness in the four groups besides the BC group, the treatment will be conducted in a separate room or a curtain will be used to cover the bed, while patients will be wearing an eye mask. Third, to improve compliance and reduce the rate of loss to follow-up, the researchers will remind the patient 1 day before the examination, and the patient will be accompanied by a special person.

Although there are many difficulties associated with this study, we will strive to conduct research according to the research norms and to ensure the research quality meets the highest standard. This is the first clinical observation using facial nerve color ultrasound to explore the potential therapeutic effect and the recovery of facial nerve edema of different acupuncture modalities on patients with Bell’s palsy in the acute phase and to initially explore its possible mechanisms. The results of this study will help to provide novel visual evidence for applications of different acupuncture modalities in the treatment of patients with Bell’s palsy in the acute phase.

## Ethics statement

The studies involving humans were approved by evidence-based capacity building project for basic Chinese medicine. The studies were conducted in accordance with the local legislation and institutional requirements. The participants provided their written informed consent to participate in this study. Written informed consent was obtained from the individual(s) for the publication of any potentially identifiable images or data included in this article.

## Author contributions

ZW: Investigation, Writing – original draft. JZ: Funding acquisition, Software, Writing – review & editing. ZZ: Investigation, Methodology, Visualization, Writing – review & editing. YL: Investigation, Methodology, Writing – review & editing. SR: Project administration, Supervision, Writing – review & editing. HS: Data curation, Formal analysis, Writing – review & editing. DM: Investigation, Software, Writing – review & editing. RL: Investigation, Validation, Writing – review & editing. YZ: Conceptualization, Project administration, Writing – original draft.

## Glossary

SFGS: Sunnybrook facial grading system
HBGS: House-Brackmann facial nerve grading system
MRI: Magnetic resonance imaging
RCT: Randomized controlled trial
CMU1H: First Affiliated Hospital of China Medical University
TCM: Traditional Chinese medicine
CONSORT: Consolidated Standards of Reporting Trials
STRICTA: Standards for Reporting Intervention in Controlled Trials of Acupuncture
A: Acupuncture
EA: Electroacupuncture
SA: Sham acupuncture
BC: Blank control
AC: Acupuncture control
AAN: American Academy of Neurology
AAO-HNSF: American Academy of Otolaryngology-Head and Neck Surgery Foundation
Vs: Systolic peak velocity
Vd: End-diastolic velocity
RI: Resistance index
IL-6: Interleukin-6
IL-8: Interleukin-8
IL-10: Interleukin-10
TNF-α: Tumor necrosis factor-α
CRF: Case Report Form
SD: Standard deviation
ANCOVA: Analysis of covariance
SOP: Standard operating procedures
NLR: Neutrophil-to-lymphocyte
SFORL: French Society of ENT and Head and Neck Surgery
