# Supplementary material for: Effects of acupuncture for Bell’s palsy patients in the acute phase and its impact on facial nerve edema: a study protocol for a randomized, controlled trial
Source: Front Neurol. 2024 Apr 16;15:1327206. doi: 10.3389/fneur.2024.1327206 (PMC11058209; doi:10.3389/fneur.2024.1327206)
Supplement: Supplementary file 1 [file Data_Sheet_1.doc]

**SPIRIT Checklist: Recommended Items to Address in a Clinical Trial Protocol and Related Documents**

| **Section/Item**  **Administrative information** | **Item Number** | **Description** |
| --- | --- | --- |
| Title | 1 | Descriptive title identifying the study design, population, interventions, and, if applicable, trial acronym  Effects of acupuncture for Bell’s palsy patients in the acute phase and its impact on facial nerve edema: A study protocol for a randomized,controlled trial |
| Trial registration | 2a | Trial identifier and registry name. If not yet registered, name of intended registry.  Trial identifier：chictr.org.cn: ChiCTR2100050815  Registry name: Randomized controlled trial of acupuncture in patients with acute Bell's palsy |
|  | 2b | All items from the World Health Organization Trial Registration Data Set (Appendix Table, available at [www.annals.org](http://www.annals.org))  Not applicable for this trial. |
| Protocol version | 3 | Date and version identifier  Date [September](D:/Program%20Files%20(x86)/Youdao/Dict/8.9.6.0/resultui/html/index.html#/javascript:;) 4, 2021 and version 2.0 |
| Funding | 4 | Sources and types of financial, material, and other support  This work was supported by National basic evidence-based capacity building project of traditional Chinese Medicine（[2019]130）. |
| Roles and responsibilities | 5a | Names, affiliations, and roles of protocol contributors Zhidan Wang1, Jie Zhang1, Zhen Zhang2, Yue Liu1,Shuang Ren1,Hao Sun3, Di Meng4,Ruoshi Liu1, Yang Zhang1,*  1Department of Traditional Chinese Medicine, The First Hospital of China  Medical University, Shenyang,China  2Department of Ultrasound, The First Hospital of China  Medical University, Shenyang,China  3Department of clinical epidemiology,The First Hospital of China Medical University, Shenyang, China  4Department of geratology,The First Hospital of China Medical University,Shenyang, China  Authors’contributions: WZD and ZY designed and developed the trial. WZD drafted the manuscript. ZJ and RS made key revisions to this paper. LY provided details of acupuncture technical support. ZZ provided ultrasonic technical support. SH carried out the statistical analysis.MD and LRS provided help in the literature search. All authors have read and approved the final manuscript. |
|  | 5b | Name and contact information for the trial sponsor  Science and Technology Department of the State Administration of Traditional Chinese Medicine of China,1 Gongti West Road, Dongcheng District, Beijing100027,China |
|  | 5c | Role of study sponsor and funders, if any, in study design; collection, management, analysis, and interpretation of data; writing of the report; and the decision to submit the report for publication, including whether they will have ultimate authority over any of these activities  Funders of this study have no role in any abovementioned activities. |
|  | 5d | Composition, roles, and responsibilities of the coordinating center, steering committee, end point adjudication committee, data management team, and other individuals or groups overseeing the trial, if applicable (see item 21a for DMC)  Zhidan Wang and Yang Zhang designed the described study. Yue Qi,Yunxi Li and Zhen Zhang conduct the research. Shuang Ren and Yue Liu collect data. Hao Sun and Di Meng made statistical analysis. The data and safety monitoring of this trial will be entrusted to the data monitoring committee (DMC) and the Data and Safety Monitoring Committee (DSMB) of the First Hospital of China Medical University, which are independent of the sponsors and the research group and have no competitive interests. |
| **Introduction** | | |
| Background and rationale | 6a | Description of research question and justification for undertaking the trial, including summary of relevant studies (published and unpublished) examining benefits and harms for each intervention  Bell’s palsy is an acute peripheral facial neuropathy, which is one of the most common causes of facial palsy of lower motor neurons. Facial nerve swelling is commonly observed in Bell’s palsy. Acupuncture therapy has been  widely used in the treatment of Bell’s palsy. However, whether acupuncture can be effectively used in the acute stage is still controversial. There are no clinical trials conducted previously to evaluate the effect of acupuncture on facial nerve edema in Bell’s palsy patients. The study aims to evaluate the potential efficacy of different acupuncture modalities on Bell’s palsy patients in the acute phase, its effect on facial nerve edema, and to preliminarily explore its possible mechanism. |
|  | 6b | Explanation for choice of comparators  We choose sham acupuncture(sham acupuncture+medicine),blank control(pure medicine)and acupuncture control(pure Acupuncture0 as the comparators in this study. |
| Objectives | 7 | Specific objectives or hypotheses  The objectives of this trial are:  (1) To evaluate the efficacy of different acupuncture modalities on patients with Bell’s palsy in the acute stage;  (2) To assess whether different acupuncture modalities can effectively relieve facial nerve edema in patients with Bell’s palsy in the acute stage;  (3) To analyze whether different acupuncture modalities can significantly improve facial blood circulation and muscle activity in patients with Bell’s palsy in the acute stage;  (4) To provide reference for the timing of different acupuncture modalities intervention;  (5) To explore the mechanisms that can contribute to both inflammatory and immune responses. |
| Trial design | 8 | Description of trial design, including type of trial (e.g., parallel group, crossover, factorial, single group), allocation ratio, and framework (e.g., superiority, equivalence, noninferiority, exploratory)  This study is a single-center, randomized, controlled clinical trial carried out in the First Hospital of China Medical University. |
| **Methods** | | |
| Participants, interventions, and outcomes | | |
| Study setting | 9 | Description of study settings (e.g., community clinic, academic hospital) and list of countries where data will be collected. Reference to where list of study sites can be obtained  It will be conducted from October 2021 to December 2025 in the Traditional Chinese Medicine(TCM) Department, Emergency Department, Ultrasound Department, and Neurology Department of CMU1H, which is the largest comprehensive hospital in Northeast China.（located at No. 155, Nanjing North Street, Heping District, Shenyang 10001）. |
| Eligibility criteria | 10 | Inclusion and exclusion criteria for participants. If applicable, eligibility criteria for study centers and individuals who will perform the interventions (e.g., surgeons, psychotherapists)  Inclusion criteria   1. Aged 18-70 years; 2. Meets the diagnostic criteria for Bell’s palsy, HBGS should be graded from Ⅱ to VI, and the total score of SFGS should be ≤ 89; 3. Onset is less than or equal to 3 days; 4. Unilateral onset; 5. Agree to participate in the investigation and sign the written informed consent.   Exclusion criteria  Participants who will meet any of the following conditions will be excluded:   1. Participants who have otitis media, mastoiditis, labyrinthinitis, mumps and other complications of the peripheral facial paralysis; 2. Participants who have a prior diagnosis of the [peripheral](D:/Program%20Files%20(x86)/Youdao/Dict/8.9.6.0/resultui/html/index.html#/javascript:;) [facial](D:/Program%20Files%20(x86)/Youdao/Dict/8.9.6.0/resultui/html/index.html#/javascript:;) [paralysis](D:/Program%20Files%20(x86)/Youdao/Dict/8.9.6.0/resultui/html/index.html#/javascript:;) caused by posterior fossa lesions such as acoustic neuroma, skull base meningitis, intracranial metastasis of cancer, multiple sclerosis, etc; 3. Participants diagnosed with the central facial paralysis; 4. Participants diagnosed with [Hunt's](D:/Program%20Files%20(x86)/Youdao/Dict/8.9.6.0/resultui/html/index.html#/javascript:;) [syndrome](D:/Program%20Files%20(x86)/Youdao/Dict/8.9.6.0/resultui/html/index.html#/javascript:;); 5. Participants with serious cardiovascular and cerebrovascular diseases, diabetes, hypertension, serious primary diseases of the liver, kidney, lung and blood system, malignant tumor, ulcer of digestive system and bleeding tendency are expected to fail to complete the test; 6. Participants who might be enrolled in other clinical trials within 1 month; glaucoma patients; pregnant or lactation women or severe allergic conditions. Those who have neurological, mental illness, illiteracy or poor compliance in the screening process will not be eligible to fill in the questionnaire. |
| Interventions | 11a | Interventions for each group with sufficient detail to allow replication, including how and when they will be administered  The interventions will be combined with the guidelines published by the American Academy of Neurology (AAN) and the American Academy of Otolaryngology-Head and Neck Surgery Foundation (AAO-HNSF) in 2013 and the preliminary experiments of this study. Acupuncture will be performed by three different experienced acupuncturists in accordance with STRICTA. The subjects in each group except the blank control group will receive either acupuncture, electroacupuncture or sham acupuncture treatment five times per week for four weeks. With the exception of the acupuncture control group, patients in each group will receive the following medications. Prednisone acetate tablets will be administered orally 30 mg per day for 5 days, then it will be reduced by 5 mg per day until discontinued for 10 days (Sinopharm, Rongsheng Pharmaceutical Co. LTD., Jiaozuo, China). Mecobalamin tablets 0.5mg orally, 3 times a day (Eisai (China) Pharmaceutical Co., LTD., Jiaozuo, China).Vitamin B1 tablets 10mg orally 3 times a day (Fuzhou Haiwang Fuyao Pharmaceutical Co., LTD., Fuzhou, China).During the study, subjects will not be allowed to use other traditional Chinese medicine treatment methods other than those prescribed medications for the treatment of the disease (e.g. Traditional Chinese medicine preparations, tuina therapy, etc.).  Acupuncture (A) group and acupuncture control (AC) group  The disposable sterile acupuncture needles will be used (0.25 × 40 mm, Suzhou Acupuncture supplies Co., LTD., China). In all sessions, patients will be treated with 8 core acupuncture points and 4 additional acupoints. The core acupoints will be BL02 (Cuanzhu), GB14 (Yangbai), ST2 (Sibai), SI18 (Quanliao), ST4 (Dicang), ST6 (Jiache),LI4 (Hegu), LR3 (Taichong), and the additional acupoints can be chosen from the following points: middle ditch askew plus DU26 (Shuigou); nasolabial fold becomes shallow add LI20 (Yingxiang); eyelid closure difficulty plus EX-HN5 (Taiyang); pain behind ear mastoid process Ex-HN17 (Yifeng). All acupoints will be carefully selected according to the clinical experience and the literature. After the acupuncture operation, the needle will be retained for 30 min before removal. The treatment will be performed once a day, 5 times as a course of treatment, with 2d rest between the courses, for a total of 4 courses. Acupuncture group will be combined with medicine therapy on the basis of acupuncture treatment. The acupuncture control group will be treated with acupuncture only. The acupuncture points and methods have been summarized in Table2.  Electroacupuncture (EA) group  The acupuncture points and treatment course of this group will be the same as those of group A and AC, and 4 acupoints will be connected to an electroacupuncture device and treated with electroacupuncture. The needles on GB14 (Yangbai), SI18(Quanliao), ST4 (Dicang)and ST6 (Jiache) will be connected to a SDZ-Ⅱ Acupuncture Stimulating Instruments (Suzhou Medical Supplies Factory Co., LTD., China) with 2-Hz frequency and the varying amplitude according to the comfort of the participants, ranging from 2-5 mA to enhance the sensation of acupuncture. The needles will be kept for 30 min and then removed.  Sham acupuncture (SA) group  Patients in this group will be treated with sham acupuncture at the following 5 non-meridian and non-acupoint stimulation points, and the specific location has been shown in Table 3. The location of acupoints can be avoided by referring to the 2006 National standard of the People's Republic of China (GB/T 12346-2006) name and location of acupoints. Patients will be placed in supine position, their skin will be disinfected, and Streitberger Placebo-needle (0.30 × 30 mm) will be used [25]. When the needle will be attached to the skin through a plastic ring, the patient might feel a tingling sensation, mimicking a puncture to the skin. However, when the needle is pressed against the skin, it does not penetrate the skin but instead retracts into the handle. The frequency, course and time of acupuncture will be the same as those of acupuncture group.  Blank control (BC) group  Except for medicine therapy, no electropuncture, acupuncture or sham acupuncture will be carried out during the whole experiment in this group. |
|  | 11b | Criteria for discontinuing or modifying allocated interventions for a given trial participant (e.g., drug dose change in response to harms, participant request, or improving/worsening disease)  The participants can withdraw from the trial for any reason and at any time. Researchers can remove participants from the trial with any of the following conditions may be removed from the study: 1) The legal representative of the patient requested to withdraw from the study; 2) Serious adverse events;3)Death. |
|  | 11c | Strategies to improve adherence to intervention protocols, and any procedures for monitoring adherence (e.g., drug tablet return, laboratory tests)  Investigators experimenting will be trained before the start. The subjects will undergo daily facial function assessments through SFGS and HBGS, and blood will be drawn at fixed times for laboratory testing.At the same time,the investigator and data collector will remind the subjects about this procedure one day prior to the scheduled visit date. |
|  | 11d | Relevant concomitant care and interventions that are permitted or prohibited during the trial  During the study, subjects will not be allowed to use other traditional Chinese medicine treatment methods other than those prescribed medications for the treatment of the disease (e.g. Traditional Chinese medicine preparations, tuina therapy, etc.). |
| Outcomes | 12 | Primary, secondary, and other outcomes, including the specific measurement variable (e.g., systolic blood pressure), analysis metric (e.g., change from baseline, final value, time to event), method of aggregation (e.g., median, proportion), and time point for each outcome. Explanation of the clinical relevance of chosen efficacy and harm outcomes is strongly recommended  Primary outcomes  The primary outcome is the effectiveness rate after the intervention period, which will be evaluated immediately at the end of treatment on the 20 th day. Combined with the results of SFGS and HBGS, the efficacy evaluation criteria have been formulated according to the Evaluation and efficacy standard of Integrated Chinese and Western Medicine for peripheral facial nerve palsy (draft) by Yang et al. as shown in Table 4. The effectiveness rate is calculated by dividing the sum of the number of cured, efficacious, and effective patients in each group by the number of patients in each group. The Sunnybrook facial grading system (SFGS) is composed of three distinct areas that generate a comprehensive score describing the overall static and dynamic state of the face. Final score=free movement points-static points-linkage points. SFGS score can range between 0 and 100, and the higher the score is, the better facial nerve function . The House-Brackmann facial nerve grading system (HBGS) is a scale for assessing the severity of Bell’s palsy, which can potentially classify facial nerve injury into six grades . Grade I indicates normal function, grade II mild dysfunction, grade≤IV moderate palsy, and grade≥V severe palsy (28). SFGS and HBGS scores will be evaluated prior to each treatment until the end of the study and during the follow-up periods of 8 and 12weeks of treatment.   - Recovery speed: The patient's recovery will be evaluated daily through SFGS and HBGS until the patient is assessed as “cure” ,according to the evaluation criteria for curative effect(Table 4).The period from baseline to this time is considered the recovery time.If the patient's recovery time is shorter, it will be considered that the recovery speed is faster. - Diameter of facial nerve: Bilateral nerve color Doppler ultrasonography will be conducted by a board-certified neurosonologist in the Department of Ultrasound of CMU1H. The diameter of the main trunk and 5 branches of facial nerve on both sides of the face will be measured for 3 times, and the average value will be taken. - Echo intensity and thickness of facial muscles: The thickness and recovery strength of frontalis muscle, orbicularis oculi muscle, orbicularis oris muscle, depressor anguli oris muscle, depressor labii inferioris and mentalis muscle will be measured using diagnostic ultrasound system. The determination of maximum muscle thickness has been reported to be orthogonal to the direction of muscle fibers [20]. Thus, gray analysis will be used to quantify the echo intensity of each muscle [20]. The results of three independent measurements for each muscle will be averaged to minimize the differences. - Blood flow parameters of facial artery: Color Doppler ultrasound will be used to measure and record the systolic peak velocity (Vs), end-diastolic velocity (Vd) and resistance index (RI) of bilateral [facial](D:/Program%20Files%20(x86)/Youdao/Dict/8.9.6.0/resultui/html/index.html#/javascript:;) [artery](D:/Program%20Files%20(x86)/Youdao/Dict/8.9.6.0/resultui/html/index.html#/javascript:;), inferior labial [artery](D:/Program%20Files%20(x86)/Youdao/Dict/8.9.6.0/resultui/html/index.html#/javascript:;) and superior labial [artery](D:/Program%20Files%20(x86)/Youdao/Dict/8.9.6.0/resultui/html/index.html#/javascript:;). All data will be measured for 3 times and thereafter averaged - Serum inflammatory level : At the baseline, day 5 of treatment, day 10 of treatment, day 20 of treatment, week 8 of treatment and week 12 of treatment, the count of neutrophils, lymphocytes, platelets and the levels of interleukin-6 (IL-6), interleukin-8 (IL-8), interleukin-10 (IL-10) and tumor necrosis factor-α (TNF-α) will be measured to assess the levels of immune inflammation. - Safety evaluation :The various safety indicators will be measured at the baseline and at the end of treatment, including blood routine(the count of vneutrophils, lymphocytes, platelets,monocytes,Hemoglobin content), liver function (Alanine aminotransferase,Aspartate aminotransferase)and kidney function(Blood urea nitrogen,Serum creatinine). - Adverse events:Acupuncture can exhibit potential adverse events, such as pain, hematoma and infection, and the participants will be clearly informed about all these adverse events before signing informed consent. We will also provide appropriate medical care for the commonly observed adverse reactions. Any adverse events will be recorded by CRF. In case of serious adverse events, treatment will be terminated and a detailed report will be made to researchers and the ethics committee within 24 hours after the occurrence. The ethics committee will make recommendations and decide whether the patient can continue the treatment. We will give them proper compensation for their medical expenses. |
| Participant timeline | 13 | Time schedule of enrollment, interventions (including any runins and washouts), assessments, and visits for participants. A schematic diagram is highly recommended (Figure).  As shown in Figure 1 and Table 1. |
| Sample size | 14 | Estimated number of participants needed to achieve study objectives and how it was determined, including clinical and statistical assumptions supporting any sample size calculations  The calculation of sample size is based on the effective rate of acupuncture in the treatment of patients with Bell’s palsy in the acute stage. According to the previous studies and pre-experimental analysis of the research group, it is estimated that the effective rate of EA group is 99%, that of A group is 95%, that of AC group is 85%,that of SA group is 74%, and that of BC group is 70%. We have used pass 15 software to calculate and set α= 0.05, β= 0.1, and the results showed that the total sample size was at least 147 cases. It was observed that based on the ratio of sample size 1:1:1:1:1 in each group and considering the loss of follow-up rate of 10%, an optimal sample size of 33 participants should be recruited in each group. Therefore, a total of 165 participants should be recruited for this RCT. |
| Recruitment | 15 | Strategies for achieving adequate participant enrollment to reach target sample size  Close collaboration between physicians and researchers in every Departments. Each potential subject will be informed to the researcher to initiate the standard recruitment protocol. |
| Assignment of interventions (for controlled trials) | | |
| Allocation Sequence generation | 16a | Method of generating the allocation sequence (e.g., computer-generated random numbers), and list of any factors for stratification. To reduce predictability of a random sequence, details of any planned restriction (e.g., blocking) should be provided in a separate document that is unavailable to those who enroll participants or assign interventions.  A random sequence will be generated based on the block randomization by an independent research assistant using the software SPSS 26.0. The various eligible participants will be randomly divided into 5 groups with 1:1:1:1:1 ratio. Randomization assignment will be carried out by a researcher(Ji Qi) not involved in the treatment and assessment. |
| Allocation concealment mechanism | 16b | Mechanism of implementing the allocation sequence (e.g., central telephone; sequentially numbered, opaque, sealed envelopes), describing any steps to conceal the sequence until interventions are assigned  The processing assignment codes will be encapsulated in the sequentially numbered opaque envelopes by an independent researcher. |
| Implementation | 16c | Who will generate the allocation sequence, who will enroll participants, and who will assign participants to interventions  The researcher(Ji Qi) not involved in the treatment and assessment will generate the allocation sequence, Yue Qi and Yunxi Li will enroll participants and assign participants to interventions according to pre-prepared allocation sequence. |
| Blinding (masking) | 17a | Who will be blinded after assignment to interventions (e.g., trial participants, care providers, outcome assessors, data analysts), and how  The laboratory technicians, ultrasound doctors, and statisticians will be blinded to the treatments, with no unblinding needed under any condition.At the same time, this trial will be blinded to recruited patients, the acupuncturists, data collectors, assessor, and statisticians between the other four groups. |
|  | 17b | If blinded, circumstances under which unblinding is permissible, and procedure for revealing a participant’s allocated intervention during the trial  Patients will be treated in separate room or a curtain will be used to cover the bed, while patients will be wearing an eye mask. In the sham acupuncture group, the acupoints will be on the face and back of limbs, and the operation experience will consistent with that of acupuncture. |
| Data collection, management, and analysis | | |
| Data collection methods | 18a | Plans for assessment and collection of outcome, baseline, and other trial data, including any related processes to promote data quality (e.g., duplicate measurements, training of assessors) and a description of study instruments (e.g., questionnaires, laboratory tests) along with their reliability and validity, if known. Reference to where data collection forms can be found, if not in the protocol.  The participants involved in the implementation will be trained in a unified manner, with unified recording methods and judgment standards. We will formulate and implement rigorous, detailed, and feasible relevant standard operating procedures (SOP), and the clinical supervisors as well as the data supervisors will carefully supervise the whole process of investigation according to the SOP. The investigator shall accurately and carefully record all the contents in Case report form (CRF) in accordance to the filling requirements of CRF, to ensure the authenticity and reliability of CRF. All observations and findings in the trial will be reviewed to ensure the reliability of the data and to ensure that all conclusions in the clinical trial have been derived from the original data. |
|  | 18b | Plans to promote participant retention and complete follow-up, including list of any outcome data to be collected for participants who discontinue or deviate from intervention protocols  Follow-up phone call will be conducted to complete follow-up.At the same time,the investigator and data collector will remind the subjects about this procedure one day prior to the scheduled visit date. |
| Data management | 19 | Plans for data entry, coding, security, and storage, including any related processes to promote data quality (e.g., double data entry; range checks for data values). Reference to where details of data management procedures can be found, if not in the protocol.  There will be two data administrators entering and proofreading the data to ensure accuracy. If any problem is detected with the data, the data supervisor will ask the researcher for clarification. After the study, the clinical researchers, data managers, and statistical analysts will carefully review the established database. After a blind audit and confirmation of the correctness of the established database, principal researchers and statistical analysts will lock the data. The locked data files will remain unchanged. The data will only be used for this specific research project. |
| Statistical methods | 20a | Statistical methods for analyzing primary and secondary outcomes. Reference to where other details of the statistical analysis plan can be found, if not in the protocol.  The statistical analysis of this trial will use SPSS 26.0 software. To include the data of the participants who might withdraw later from the trial, all statistical analyses were based on the intention-to-treat population of all randomly assigned patients. Missing data will be analyzed using multiple imputations using the Markov chain  Monte Carlo. The continuity data will be described as mean±standard deviation (SD) or median and interquartile spacing. The classified data will be described with the frequency and percentage (N, %). The assessment of the difference between groups will use repeated measure analysis of covariance (ANCOVA) and trend chi-square, and the mixed effect model will also be used to evaluate the efficacy based on adjusting possible covariates. For results that require a comparison between multiple groups, Bonferroni will be used for multiple corrections. An interim analysis will be performed when half of the participants have completed the main outcome measurement. |
|  | 20b | Methods for any additional analyses (e.g., subgroup and adjusted analyses)  Not applicable for this trial. |
|  | 20c | Definition of analysis population relating to protocol nonadherence (e.g., as-randomized analysis), and any statistical methods to handle missing data (e.g., multiple imputation)  Missing data will be analyzed by multiple imputation. |
| Monitoring | | |
| Data monitoring | 21a | Composition of DMC; summary of its role and reporting structure; statement of whether it is independent from the sponsor and competing interests; and reference to where further details about its charter can be found, if not in the protocol. Alternatively, an explanation of why a DMC is not needed.  The data and safety monitoring of this trial will be entrusted to the data monitoring committee (DMC) and the Data and Safety Monitoring Committee (DSMB) of the First Hospital of China Medical University, which are independent of the sponsors and the research group and have no competitive interests. |
|  | 21b | Description of any interim analyses and stopping guidelines, including who will have access to these interim results and make the final decision to terminate the trial  The primary role of DMC will be to monitor the treatment and integrity of the whole trial data, and conduct interim analysis to confirm whether the trial complies with the principles of this protocol. DSMB is composed of five renowned experts in the different fields and monitors the performance as well as the safety of the trial every 6 months. It will have the right to obtain the various interim results of the trial, reveal the participant’s allocated intervention and take the the final decision regarding the termination of the trial. |
| Harms | 22 | Plans for collecting, assessing, reporting, and managing solicited and spontaneously reported adverse events and other unintended effects of trial interventions or trial conduct  Acupuncture can exhibit potential adverse events, such as pain, hematoma and infection, and the participants will be clearly informed about all these adverse events before signing informed consent. We will also provide appropriate medical care for the commonly observed adverse reactions. Any adverse events will be recorded by CRF. In case of serious adverse events, treatment will be terminated and a detailed report will be made to researchers and the ethics committee within 24 hours after the occurrence. The ethics committee will make recommendations and decide whether the patient can continue the treatment. We will give them proper compensation for their medical expenses. |
| Auditing | 23 | Frequency and procedures for auditing trial conduct, if any, and whether the process will be independent from investigators and the sponsor  DSMB is composed of five renowned experts in the different fields and monitors the performance as well as the safety of the trial every 6 months,which are independent of the sponsors and the research group and have no competitive interests. |
| **Ethics and dissemination** | | |
| Research ethics approval | 24 | Plans for seeking REC/IRB approval  The study protocol has been approved by the Ethics Committee of The First Affiliated Hospital of China Medical University ([2021]326). |
| Protocol amendments | 25 | Plans for communicating important protocol modifications (e.g., changes to eligibility criteria, outcomes, analyses) to relevant parties (e.g., investigators, RECs/IRBs, trial participants, trial registries, journals, regulators)  Any modifications to the protocol will require a formal application to Ethics Committee of the First Hospital of China Medical University, as well as to the Chinese clinical trial registry. |
| Consent or assent | 26a | Who will obtain informed consent or assent from potential trial participants or authorized surrogates, and how (see item 32)  Yue Qi and Yunxi Li will obtain informed consent from potential trial participants. |
|  | 26b | Additional consent provisions for collection and use of participant data and biological specimens in ancillary studies, if applicable  Not applicable for this trial. |
| Confidentiality | 27 | How personal information about potential and enrolled participants will be collected, shared, and maintained in order to protect confidentiality before, during, and after the trial  The patient recruited will receive a participation ID in this study, with which personal information of participants is labeled in the chart.The principal researchers as well as statistical analysts will lock the data. The locked data files will remain unchanged. The data will only be used for the purpose of this specific research project. The electronic data files will be password protected and only the principal investigator will have access to all the files. |
| Declaration of interests | 28 | Financial and other competing interests for principal investigators for the overall trial and each study site  None. |
| Access to data | 29 | Statement of who will have access to the final trial data set, and disclosure of contractual agreements that limit such access for investigators  Jie Zhang and Yang Zhang are responsible for the data and have the final dataset. |
| Ancillary and post-trial care | 30 | Provisions, if any, for ancillary and post-trial care, and for compensation to those who suffer harm from trial participation  Not applicable. |
| Dissemination policy | 31a | Plans for investigators and sponsor to communicate trial results to participants, health care professionals, the public, and other relevant groups (e.g., via publication, reporting in results databases, or other data-sharing arrangements), including any publication restrictions  Upon reasonable request, the data sets generated and/or analyzed can be obtained from the corresponding authors. The results of this study will be published in open-access and peer-reviewed journals. |
|  | 31b | Authorship eligibility guidelines and any intended use of professional writers  We haven’t used such a service. |
|  | 31c | Plans, if any, for granting public access to the full protocol, participant-level data set, and statistical code  This was already mentioned in 18a and 31a. |
| **Appendices** | | |
| Informed consent materials | 32 | Model consent form and other related documentation given to participants and authorized surrogates  Model consent form and other related documentation given to participants and authorized surrogates are available from the corresponding author on reasonable request. |
| Biological specimens | 33 | Plans for collection, laboratory evaluation, and storage of biological specimens for genetic or molecular analysis in the current trial and for future use in ancillary studies, if applicable  Blood samples from participants will be collected and stored at -80℃ for the final analysis. |
